# Modeling the Amplification of Immunoglobulins through Machine Learning on Sequence-Specific Features

**DOI:** 10.1038/s41598-019-47173-w

**Published:** 2019-07-24

**Authors:** Matthias Döring, Christoph Kreer, Nathalie Lehnen, Florian Klein, Nico Pfeifer

**Affiliations:** 1 Department of Computational Biology and Applied Algorithmics, Max Planck Institute for Informatics, Saarland Informatics Campus, 66123 Saarbrücken, Germany; 2 Institute of Virology, University of Cologne, Fürst-Pückler-Str. 56, 50935 Cologne, Germany; 3 Center for Molecular Medicine, University Hospital of Cologne, Robert-Koch-Straße 21, 50931 Cologne, Germany; 4 German Center for Infection Research, Cologne-Bonn Partner Site, Cologne, Germany; 5 Methods in Medical Informatics, Department of Computer Science, University of Tübingen, Sand 14, 72076 Tübingen, Germany; 6 Medical Faculty, Geissweg 5, University of Tübingen, 72076 Tübingen, Germany; 7 German Center for Infection Research, Tübingen Partner Site, Tübingen, Germany

**Keywords:** PCR-based techniques, Computational models, Software, Machine learning

## Abstract

Successful primer design for polymerase chain reaction (PCR) hinges on the ability to identify primers that efficiently amplify template sequences. Here, we generated a novel *Taq* PCR data set that reports the amplification status for pairs of primers and templates from a reference set of 47 immunoglobulin heavy chain variable sequences and 20 primers. Using logistic regression, we developed TMM, a model for predicting whether a primer amplifies a template given their nucleotide sequences. The model suggests that the free energy of annealing, Δ*G*, is the key driver of amplification (p = 7.35e-12) and that 3′ mismatches should be considered in dependence on Δ*G* and the mismatch closest to the 3′ terminus (p = 1.67e-05). We validated TMM by comparing its estimates with those from the thermodynamic model of DECIPHER (DE) and a model based solely on the free energy of annealing (FE). TMM outperformed the other approaches in terms of the area under the receiver operating characteristic curve (TMM: 0.953, FE: 0.941, DE: 0.896). TMM can improve primer design and is freely available via openPrimeR (http://openPrimeR.mpi-inf.mpg.de).

## Introduction

Polymerase chain reaction (PCR) forms the foundation for a multitude of a variety of molecular methods (e.g. determining drug resistance^1,2^ and viral loads^3^). Primers – short nucleotide oligomers complementary to template DNA – are critical for the effective amplification of templates through PCR. For example, the optimization of primers targeting immunoglobulin variable gene sequences is critical for the identification of novel antibodies such as broadly neutralizing antibodies targeting HIV-1^4^. Models that estimate PCR efficiencies can guide primer design for quantitative PCR (qPCR)^5–8^, while models estimating the likelihood of amplification can guide primer design for conventional PCR^9^. These models need to consider the two consecutive molecular interactions that determine whether a primer allows for the amplification of a PCR template. In the first reaction, the primer anneals to the template to form the primer-template heteroduplex. In the second reaction, polymerase attaches to the partial heteroduplex and elongates the oligonucleotide to a complementary full-length sequence^10^.

Efficient primer annealing is largely determined by the complementarity of primer and template^11^, a characteristic that is captured by the free energy of annealing. Therefore, non-complementary bases in the nucleotide sequences of primers and templates (mismatches) should be avoided. Mismatches within the 3′ hexamer of the primer-template duplex (i.e. the terminal six nucleotides) are especially detrimental as they can disrupt polymerase binding^5,6,12–15^. The impact of 3′ mismatches increases with growing proximity to the 3′ terminus^13,15^. Moreover, the extent at which 3′ terminal mismatches decrease PCR efficiency critically depends on the type of mismatch (e.g. an A/G mismatch is substantially more detrimental than an A/C mismatch)^12,13,16–20^. To stabilize the 3′region, primers are often designed to exhibit a GC clamp^21–23^ consisting of one to three Gs or Cs at the 3′ end of the primer.

Primer binding events can be identified using thermodynamic or statistical models^24^. To our best knowledge, the thermodynamic model provided by DECIPHER^8^ (DE) is the only model that is currently available. DECIPHER incorporates empiric evidence about the impact of position- and nucleotide-specific mismatches within the last seven positions of the 3′ region. These data were gathered by measuring the elongation efficiency of *Taq* polymerase in PCRs performed with 171 primers exhibiting different binding properties. The model considers three reactions: the interaction between primer and template, unimolecular folding of the primer, and unimolecular folding of the template. Based on the underlying kinetic differential equations for these reactions, the concentrations of the considered molecular states are mechanistically computed for inferring the efficiency of PCR.

Here, we present a novel *Taq* PCR data set providing the amplification status for 47 immunoglobulin heavy-chain variable (IGHV) genes. Triplicate measurements were performed with primers from two sets. Set 1 consists of 16 forward primers that have been recently designed using openPrimeR^25^, while Set 2 is a well-established set of 4 forward primers^26^. PCR was performed for each combination of the 20 primers and 47 templates giving rise to a total of 940 triplicate measurements. In contrast to other studies investigating PCR amplification, which are largely based on qPCR, this data set provides the amplification status according to gel electrophoresis. Using statistical methods, we analyzed the data set with three goals in mind. First, to investigate which physicochemical properties of primer-template pairs (PTPs) exert the greatest influence on the PCR amplification status. Second, to develop a new logistic regression model for predicting the amplification of a template. Third, to compare available models for determining amplification events.

## Results

**Figure 1:**
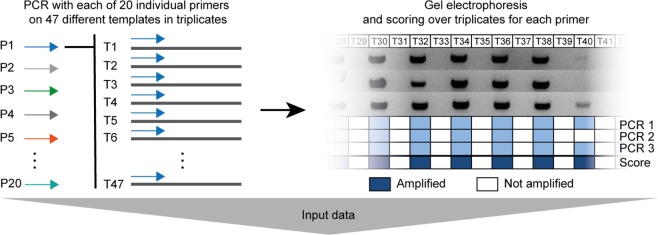
Experimental layout and labeling of the PCR reactions.

**Figure 2:**
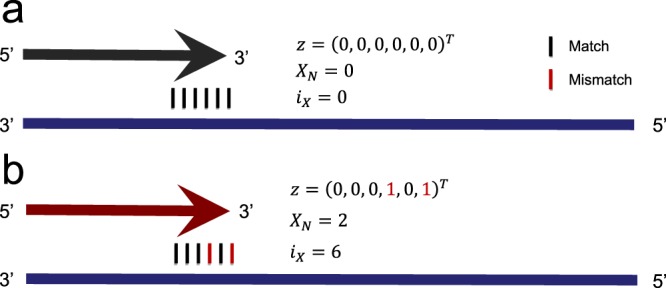
Examples for encoding mismatches within the 3′ hexamer region. Primers are indicated as arrows and templates are indicated as horizontal bars. Arrowheads indicate the 3′ hexamer region. Mismatches within the 3′ hexamer are encoded via *z* ∈ {0, 1}^6^, ${X}_{N}\in {{\mathbb{N}}}_{0}$, and *i*_*X*_ ∈ {0, 1, …, 6}. While *z* uses a binary encoding to indicate the presence of mismatches within the 3′ hexamer, *X*_*N*_ gives the total number of 3′ hexamer mismatches, and *i*_*X*_ indicates the position of the 3′ hexamer mismatch closest to the 3′ terminus. **(a)** Absence of 3′ terminal mismatches between primer and template. **(b)** Mismatches in the 3′ hexamer at positions 4 and 6.

Having selected 908 PTPs from the PCR data set, we classified the amplification status of each PTP either as *Amplified* or *Unamplified* depending on the result of gel electrophoresis (Fig. 1). To investigate which properties of PTPs are associated with the amplification status, we computed their physicochemical properties using openPrimeR, most notably, the free energy of annealing, Δ*G* [kcal/mol], and three features related to 3′ mismatches: *z* ∈ {0, 1}^6^, ${X}_{N}\in {{\mathbb{N}}}_{0}$, and *i*_*X*_ ∈ {0, 1, …, 6} (Fig. 2). We used these features to train a logistic regression model for predicting the amplification status and validated the model by comparing its performance with that from DECIPHER and an approach relying only on Δ*G*.

### Properties of the data set

Table 1 shows the distribution of the physicochemical properties of PTPs in the data set. The primers from Set 1 and Set 2 are characterized by contrasting rates of amplification. While 165 of 188 PTPs (87.8%) in Set 2 were labeled as *Amplified*, only 217 of 720 (30.1%) observations in Set 1 set were labeled as *Amplified*. Accordingly, PTPs from Set 1 exhibited a greater number of mismatches and higher free energies. The PTPs from Set 1 had an average of 2.3 mismatches in the 3′ hexamer, while the PTPs from Set 2 had an average of 0.5 mismatches in this region. Moreover, while samples from Set 2 had a ΔG inter-quartile range (IQR) of [−8.6 kcal/mol, −5.2 kcal/mol], the samples from Set 1 were associated with a higher range of [−4.9 kcal/mol, −2.0 kcal/mol].

**Table 1:** Overview of the properties of the IGHV data set.

Property	Interpretation	Set 1	Set 2
Δ*G*	Free energy of annealing	[−4.9, −2.0]	[−8.6, −5.2]
*i*_*X*_	Mismatch closest to 3′ end	[2, 6]	[0, 1]
*X*_*N*_	Number of 3′ hexamer mismatches	[1, 3]	[0, 1]
|*GC*|	Extent of GC clamp	[1, 2]	[1, 1]
Δ*G*_*f*_	Free energy of folding [kcal/mol]	[−1.53, −0.24]	[−1.24, −0.76]
Δ*G*_*s*_	Free energy of self-dimerization [kcal/mol]	[−2.1, −0.7]	[−1.2, −0.8]
y_i_ = *Amplified*	Positive amplification status	217 of 720 (30.1%)	165 of 188 (87.8%)
$\sum _{{x}_{i}}{z}_{j},\,j=1$	Number of mismatches at the start of the 3′ hexamer	271	25
$\sum _{{x}_{i}}{z}_{j},\,j=2$	Number of mismatches at the 2^nd^ position of the 3′ hexamer	226	4
$\sum _{{x}_{i}}{z}_{j},\,j=3$	Number of mismatches at the 3^rd^ position of the 3′ hexamer	272	31
$\sum _{{x}_{i}}{z}_{j},\,j=4$	Number of mismatches at the 4^th^ position of the 3′ hexamer	246	11
$\sum _{{x}_{i}}{z}_{j},\,j=5$	Number of mismatches at the 5^th^ position of the 3′ hexamer	308	12
$\sum _{{x}_{i}}{z}_{j},\,j=6$	Number of mismatches at the 3′ terminal position	308	12

Values shown in brackets indicate the inter-quartile range of the observed values.

**Figure 3:**
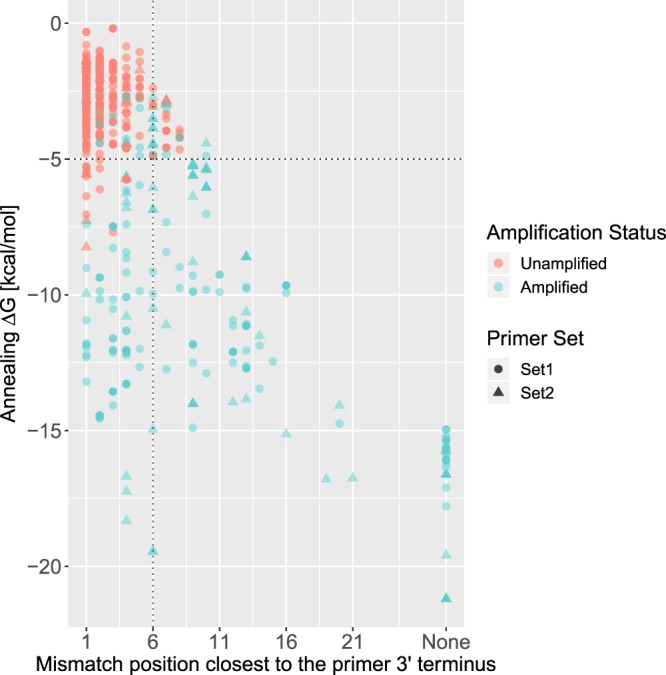
Impact of the free energy of annealing (Δ*G*) and 3′ terminal mismatches on the amplification of templates. The x-axis indicates, for every PTP, the mismatch position closest to the primer 3′ terminus such that position 1 in the plot corresponds to *i*_*X*_ = 6 and position 6 corresponds to *i*_*X*_  = 1. PTPs with zero mismatches are denoted by *None*. Every point represents a primer-template pair. Pairs that are labeled as *Amplified* are shown in blue, while those that are labeled as *Unamplified* are shown in red. Observations from Set 1 are indicated by circles and those from Set 2 by triangles. The dashed lines indicate cutoffs that are suitable for separating observations according to their amplification status. The vertical dashed line indicates the end of the 3′ hexamer, while the horizontal dashed line indicates a free energy of −5 kcal/mol.

Table 2 shows the relationship between the number of primer-template mismatches, free energy of annealing, and the rate of amplification. In our data set, primers with at most 3 mismatches had a 100% amplification rate. It is noteworthy that even primers binding with as many as 6 mismatches obtained a high amplification rate of 83.3%. Note that, for any given number of mismatches, the primers from Set 2 consistently exhibit a greater rate of amplification than the primers from Set 1. Comparing amplified and unamplified PTPs (Fig. 3), we found that the Δ*G* IQR of observations labeled as *Unamplified* was higher and more concentrated ([−2.17 kcal/mol, −1.69 kcal/mol]) than for those labeled as *Amplified* ([−12.70 kcal/mol, −5.21 kcal/mol]). Amplified samples generally exhibited fewer mismatches in the 3′ hexamer (*X*_*N*_ IQR of [0, 1] vs [2, 4]) and particularly fewer mismatches close to the 3′ terminus (*i*_*X*_ IQR of [0, 3] vs [5, 6]) than unamplified samples. Applying two-sided Wilcoxon rank-sum tests revealed that there is a significant difference between *Amplified* (*N* = 382) and *Unamplified* (*N* = 526) observations concerning both Δ*G* (p-value 1.68e-107) and *i*_*X*_ (p-value 1.51e-91).

**Table 2:** Empirical amplification rates in dependence on the number of primer-template mismatches and other properties.

Number of mismatches	*i*_*X*_	Δ*G* [kcal/mol]	Amplification rate	Primer set
0	[0, 0]	[−16.616, −15.696]	100%	Overall
1	[0, 3]	[−14.353, −12.1]	100%	Overall
2	[0, 3]	[−12.0455, −9.656]	100%	Overall
3	[0, 4]	[−11.607, −7.9185]	100%	Overall
4	[2, 6]	[−10.796, −7.409]	92.31%	Overall
5	[0, 3]	[−7.047, −6.047]	88.89%	Overall
6	[0, 0]	[−8.603, −5.11325]	83.33%	Overall
7	[0, 3]	[−5.39, −4.212]	67.19%	Overall
8	[3, 6]	[−5.56075, −2.539]	34.04%	Overall
9	[4, 6]	[−3.5335, −2.1325]	23.08%	Overall
10	[4, 6]	[−4.09, −1.724]	18.02%	Overall
11	[4, 6]	[−3.74, −1.695]	10.53%	Overall
12	[6, 6]	[−2.624, −1.413]	3.75%	Overall
0	[0, 0]	[−16.07, −15.609]	100%	Set 1
1	[0, 3]	[−13.283, −12.1]	100%	Set 1
2	[0, 3.25]	[−11.94175, −9.656]	100%	Set 1
3	[0, 4]	[−11.607, −7.66375]	100%	Set 1
4	[2, 6]	[−10.974, −6.686]	90.91%	Set 1
5	[2.5, 4.5]	[−8.36825, −6.4925]	75%	Set 1
6	[3.25, 4]	[−4.4545, −2.9]	33.33%	Set 1
7	[3, 6]	[−4.212, −2.539]	9.52%	Set 1
8	[4, 6]	[−3.303, −2.06275]	18.06%	Set 1
9	[5, 6]	[−3.0985, −2.0395]	13.51%	Set 1
10	[5, 6]	[−3.393, −1.695]	11.26%	Set 1
11	[5, 6]	[−3.351, −1.695]	4.2%	Set 1
12	[6, 6]	[−2.608, −1.413]	2.6%	Set 1
0	[0, 0]	[−20.79275, −16.616]	100%	Set 2
1	[0, 2]	[−17.782, −14.045]	100%	Set 2
2	[0, 0]	[−14.4805, −12.5605]	100%	Set 2
3	[1, 1]	[−10.505, −10.505]	100%	Set 2
4	[0.75, 2.25]	[−10.29475, −9.29225]	100%	Set 2
5	[0, 0]	[−6.047, −6.047]	100%	Set 2
6	[0, 0]	[−8.603, −5.208]	100%	Set 2
7	[0, 0]	[−5.39, −5.208]	95.35%	Set 2
8	[0, 0]	[−5.937, −3.95]	86.36%	Set 2
9	[1, 6]	[−5.58, −2.89]	78.95%	Set 2
10	[0, 3]	[−5.208, −2.956]	66.67%	Set 2
11	[0, 2.25]	[−5.208, −2.8395]	64.29%	Set 2
12	[4, 5.5]	[−2.6225, −1.9615]	33.33%	Set 2

Amplification properties are shown when evaluated on primers from all primer sets as well as on primers from Set 1 or Set 2 only, respectively.

### Logistic regression models

We used logistic regression in order to identify the features that are predictive of successful PCR amplification events. Since considered primers shared similar physicochemical properties (Table 3), we only considered properties relating to PTPs when defining the two logistic regression models *LR*_1_ and *LR*_2_ (Table 4). *LR*_1_ was defined using the features *z*, *X*_*N*_, and Δ*G*. For *LR*_2_, a term modeling the 3′ mismatch closest to the 3′ terminus, *i*_*X*_, and a term Δ*Gi*_*x*_ modeling the interaction of Δ*G* and *i*_*x*_ were additionally included. Since *LR*_1_ was not corrected for the association between Δ*G* and *i*_*X*_, only *z*_6_ (p = 8.25e-08**)** and Δ*G* (p < 2e-16) were found to be significantly predictive of the amplification status. Based on *LR*_2_, on the other hand, only Δ*G* (p = 1.78e-11) and Δ*Gi*_*x*_ (p = 5.12e-05) were found to be significantly predictive of the amplification status. This finding indicates that mismatches within the 3′ hexamer are not independent predictors of the amplification status but dependent on ΔG.

**Table 3:** Primers used for performing IGHV PCRs.

Primer ID	Sequence	GC Ratio	Δ*G*_*s*_	Δ*G*_*f*_
Set 1.1	cacctgtggttcttcctcct**cc**	59.1%	−0.8	0
Set 1.2	cacctgtggttcttcctcct**gc**	59.1%	−0.8	0
Set 1.3	atggagtttgggctgagct**gg**	57.1%	−2.3	0
Set 1.4	atggagttggggctgagct**g**	60%	−2.3	0
Set 1.5	tggagttttggctgagct**ggg**	57.1%	−2.3	−0.1
Set 1.6	actttgctccacgctcct**gc**	60%	−0.3	0
Set 1.7	atggactggacctggagcat**c**	57.1%	−1.9	0
Set 1.8	atggactggacctggaggtt**cc**	59.1%	−2.1	−1.9
Set 1.9	atggactgcacctggaggat**c**	57.1%	−1.9	0
Set 1.10	atggactggacctggagggtctt**c**	58.3%	−1.9	−3.6
Set 1.11	tctgtctccttcctcatcttcct**gc**	52%	0.4	0
Set 1.12	ggactggatttggagggtcctctt**c**	56%	−2.2	−3.2
Set 1.13	gctccgctgggttttcctt**g**	60%	0.4	0
Set 1.14	tggggtcaaccgccat**cc**	66.7%	−0.7	−1.6
Set 1.15	ggcctctccacttaaaccca**gg**	59.1%	−1.9	0
Set 1.16	tggacacactttgctacacact**cc**	50%	0	0
Set 2.1	acaggtgcccactcccaggtgca**g**	66.7%	−0.8	−1.2
Set 2.2	aaggtgtccagtgtga*r*gtgca**g**	54.3%	−1.2	0
Set 2.3	cccagatgggtcctgtcccaggtgca**g**	66.7%	−1.3	−2.6
Set 2.4	caaggagtctgttccgaggtgca**g**	58.3%	−0.8	−0.3

The extent of the primer 3′ GC clamp is indicated in bold. Primers prefixed with *Set 1* indicate primers from Set 1, while those prefixed with *Set 2* refer to primers from Set 2.

**Table 4:** Comparison of logistic regression models without (*LR*_1_) and with (*LR*_2_) correction for the association between Δ*G* and *i*_*x*_, as well as TMM, which was defined using feature selection.

Feature	*LR*_1_		*LR*_2_		TMM	
	Estimate	p-value	Estimate	p-value	Estimate	p-value
Intercept	−2.86	1.56e-12*	−5.76	6.16e-08*	−5.6177	1.80e-08*
***z***_1_	−0.50	0.0.058	−0.187	0.4929	—	—
***z***_2_	−0.00	0.977	−0.144	0.6164	—	—
***z***_3_	−0.92	0.0005*	−0.424	0.1359	—	—
***z***_4_	−0.97	0.001*	−0.46	0.1340	—	—
***z***_5_	0.04	0.894	0.574	0.1085	—	—
***z***_6_	−1.57	8.25e-08*	−0.659	0.1069	—	—
***X***_***N***_	NA	NA	NA	NA	—	—
**Δ** *G*	−0.83	<2e-16*	−1.576	1.78e-11*	−1.5448	7.35e-12*
*i*_***X***_	—	—	0.400	0.0829	0.3279	0.0818
**Δ** *Gi*_***X***_	—	—	0.180	5.12e-05*	0.1837	1.67e-05*

*NAs* indicates features that could not be estimated due to singularities. Dashes indicate features that were not considered by a model. Asterisks and bold font indicate significant features. Based on an initial significance threshold of 0.05, the following multiple hypothesis testing adjusted thresholds were used (Bonferroni): 0.05/9 = 0.0056 (*LR*_1_), 0.05/11 = 0.0045 (*LR*_2_), and 0.05/4 = 0.0125.

### Evaluated models and classifiers

In order to form a generalizable logistic regression model for predicting the likelihood of amplification, features were eliminated by performing backward stepwise selection on a model trained using the features considered in *LR*_2_. The selection procedure reduced the Akaike Information Criterion (AIC) of the initial logistic regression model from 112.34 to 102.38. Besides the intercept, the following three features were selected: Δ*G*, *i*_*X*_, and the interaction term Δ*Gi*_*X*_. In the following, this logistic regression model is called the thermodynamic mismatch model (TMM).

In order to assess the predictive performance of available approaches for predicting the likelihood of PCR amplification, we considered three models: The model DE from DECIPHER^8^, a model solely based on the free energy (FE), and TMM. Besides evaluating the quantitative output of these approaches, we also evaluated the performance of classifiers corresponding to these models by calculating a cutoff based on the estimates of each model in order to classify PTPs either as *Amplified* or *Unamplified*. Two types of cutoffs were selected for each model, one optimized for overall accuracy (by maximizing Youden′s index) and another optimized for specificity (Table 5). Classifiers optimized for overall performance and classifiers optimized for high specificity are denoted by subscription of *Y* or *s*, respectively. For example, *TMM*_*s*_ denotes the high-specificity TMM classifier and *TMM*_*Y*_ denotes the TMM classifier that was optimized for overall performance.

**Table 5:** Optimized cutoffs for the considered models for predicting PCR amplification.

Model	Cutoff interpretation	Cutoff *s* for high specificity	Cutoff *Y* for overall performance
TMM	Probability of amplification ${\hat{p}}_{c}$	83.9%	46.1%
DE	Efficiency of PCR *η*_*c*_	9.71e-05	1.88e-05
FE	Free energy of annealing ${\rm{\Delta }}{G}_{c}[\frac{kcal}{mol}]$	−6.05	−4.83

The column *Cutoff interpretation* indicates the type of values on which cutoffs were applied. The column for cutoff *s* indicates the cutoff that was selected such as to ensure an empiric specificity of at least 99%. The column for cutoff *Y* indicates the cutoff that maximized Youden′s index.

### Comparison of model and classifier performance

**Figure 4:**
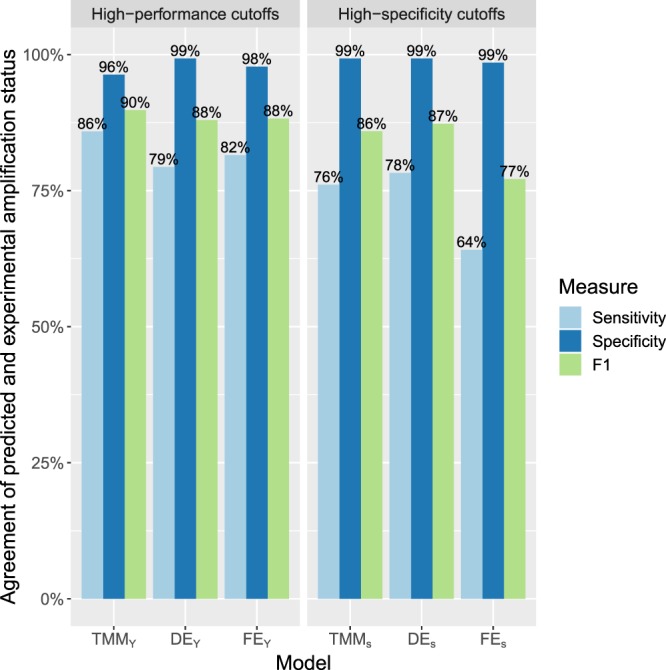
Performance of three models for identifying primer amplification events. TMM indicates our newly developed logistic regression model, DE refers to the approach from DECIPHER, and FE is solely based on the free energy of annealing. Models subscripted with *s* use cutoffs optimized for high specificity, while models subscripted with *Y* use cutoffs optimized for overall performance.

Quantitative model responses were compared with the categorical amplification status from gel electrophoresis according to the area under the receiver operating characteristic curve (AUC). TMM achieved the highest AUC (0.953) but was closely followed by FE (0.941), and DE (0.896). For all models, predictive performance was higher for observations from Set 2 than for those from Set 1 (Table 6). The classifier performance was evaluated with respect to sensitivity, specificity, and the F1 score (Fig. 4). Among high-performance classifiers, *TMM*_*Y*_ had a larger F1 score than *DE*_*Y*_ and *FE*_*Y*_ (90% vs 88% and 88%). Among high-specificity classifiers, *TMM*_*s*_ and *DE*_*s*_ outperformed *FE*_*s*_ with respect to sensitivity (76% and 78% vs 64%).

**Table 6:** Model performance in terms of the AUC when validating models on test set observations from individual primer sets.

Test set	TMM	DE	FE
Overall	0.954	0.896	0.941
Set 1	0.938	0.863	0.923
Set 2	0.980	0.941	0.980

### Interpretation of the TMM model

For interpreting and deploying TMM, a final model was trained on the full data set. The model can be specified in the following way (Table 7). Let *p* = Pr(*y*_*i*_ = *Amplified*) denote the probability that a template is amplified. Given Δ*G* and *i*_*X*_, the model estimates $\hat{p}$ = Pr(*y*_*i*_ = *Amplified*) according to its coefficients *β*_0_ = −5.62, *β*_1_ = −1.55, *β*_2_ = 0.33 and *β*_3_ = 0.18 in the following way:

$$\begin{array}{rcl}\mathrm{ln}\,\frac{\hat{p}}{1-\hat{p}} & = & {\beta }_{0}+{\beta }_{1}{\rm{\Delta }}G+{\beta }_{2}{i}_{X}+{\beta }_{3}{\rm{\Delta }}G{i}_{X}\\  & = & {\beta }_{0}+({\beta }_{1}+{\beta }_{3}{i}_{X})\cdot {\rm{\Delta }}G+{\beta }_{2}{i}_{X}\\  & = & -5.62+(-1.55+0.18{i}_{X})\cdot {\rm{\Delta }}G+0.33{i}_{X}\end{array}$$

**Table 7:** Interpretation of variables used in the formulation of the TMM model.

Term	Interpretation
$\hat{{p}}$	Estimated likelihood of amplification
ln $\frac{\hat{{p}}}{1-\hat{{p}}}$	Log odds of amplification
*β*	Model weights
**Δ** *G*	Free energy of annealing [kcal/mol]
*i*_***X***_	Position of 3′ hexamer mismatch closest to 3′ terminus of the PTP

The intercept of the model is *β*_0_ = −5.62, which indicates that the odds of template amplification are low if the other terms are negligible (i.e. for Δ*G* → 0 and *i*_*X*_ → 0). The second term, (−1.55 + 0.18 *i*_*X*_)⋅ Δ*G*, is controlled by the free energy of annealing. For typical negative values of Δ*G*, the odds of amplification increase with decreasing Δ*G* because −1.55 + 0.18 *i*_*X*_ is always negative since 0 ≤ *i*_*X*_ ≤ 6. The presence of 3′ terminal mismatches (*i*_*X*_ ≠ 0), however, reduces the odds of amplification. The third term, 0.33 *i*_*X*_, increases the odds if a 3′ mismatch is present (*i*_*X*_ ≠ 0). This term can be interpreted as a correction factor, which models that there is an overrepresentation of PTPs with high Δ*G* (e.g. −5 kcal/mol) and high *i*_*X*_.

**Figure 5:**
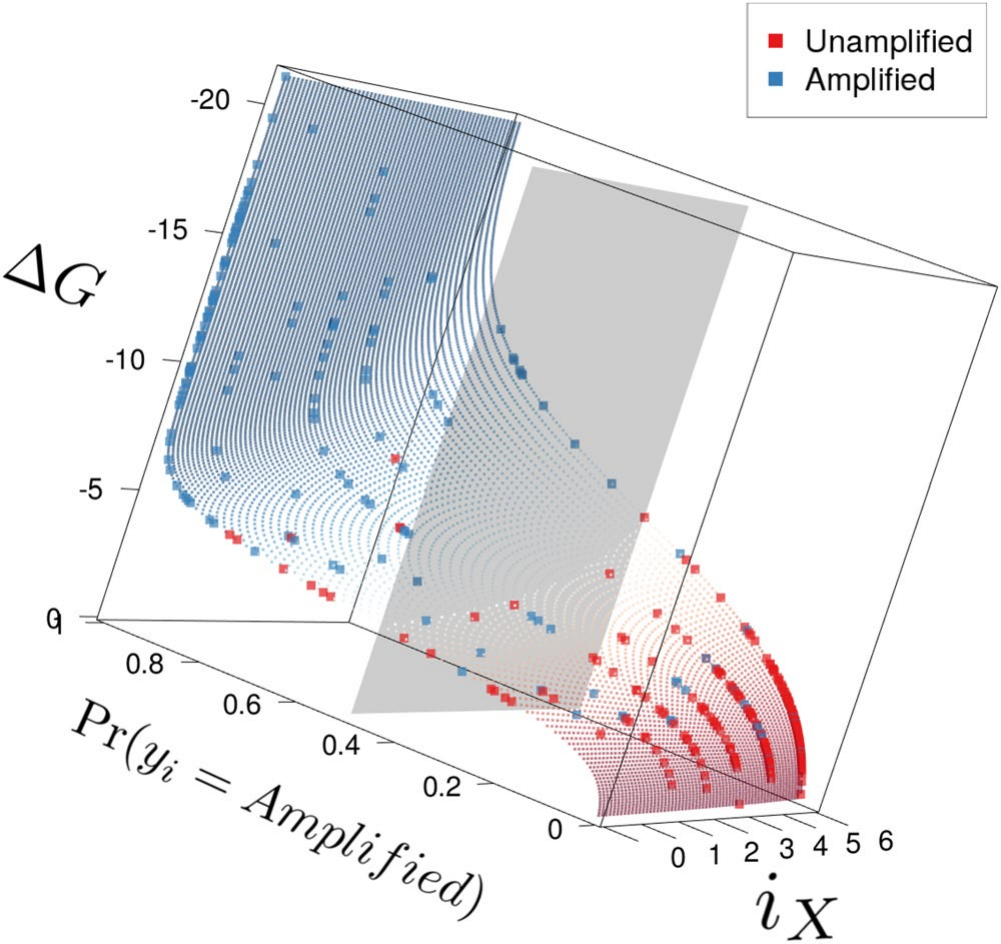
Visualization of the TMM model. Individual dots show the prediction function of the model. Red dots indicate low probabilities of amplification while blue dots indicate high probabilities. The rectangles show the model estimate for the observations contained in the data set. Here, red points indicate primer-template pairs that were labeled as *Unamplified*, while blue points indicate observations labeled as *Amplified*.

The model can be visualized as a cube (Fig. 5) whose three dimensions correspond to Δ*G*, *i*_*X*_, and the estimated likelihood of amplification, $\hat{p}$, for the PTPs in the IGHV data set. For low and high free energies (e.g. at −20 and −5 kcal/mol), Δ*G* dominates $\hat{p}$, while *i*_*X*_ influences $\hat{p}$ mostly at intermediate values of Δ*G* (e.g. at −10 kcal/mol).

## Discussion

In this work, we presented a novel PCR data set providing the amplification status for all combinations of 47 IGHV templates and 20 primers. Using these data, we investigated the interplay of the free energy of annealing and the presence of 3′ terminal mismatches and found that both factors should be considered in dependence of each other. Based on this insight, we developed TMM, a logistic regression model for predicting amplification events.

In our analysis of the IGHV data, we could mostly confirm the established factors governing the efficiency of PCR. More specifically, we could show that templates whose amplification could not be detected via gel electrophoresis are a result of primer-template conformations exhibiting high free energies, an increase in the number of mismatches within the 3′ hexamer, and a tendency for displaying mismatches close to the 3′ terminus. For the present data, however, we found that terminal mismatches by themselves are not significantly predictive of the amplification status when correcting for their association with the free energy of annealing. This finding suggests that a mismatch at the 3′ terminus does not preclude detection via gel electrophoresis as long as primer and template are otherwise highly complementary.

The newly developed TMM model for predicting amplification events has several advantages over the other models. First, since the model is based only on Δ*G* and *i*_*X*_, it is easily interpretable and it is unlikely that the model suffers from overfitting. Second, the model estimates the probability of amplification, which is a more intuitive measure than the efficiency of amplification from DE. Third, TMM achieved the largest AUC and its high-specificity classifier achieved the highest sensitivity among all classifiers. Since the present data set contains only primers exhibiting specific properties such as the absence of self-dimers and the presence of a GC clamp (Table 3), TMM neither considers primer- nor template-specific properties. Thus, it is likely that TMM overestimates the likelihood of amplification for primers exhibiting less favorable properties or when templates exhibit secondary structures^27–29^. Indeed, a previously described logistic regression model proposed by Yuryev *et al*.^9^ considered a larger number of features than TMM. Their model, however, was developed for primer genotyping assays, which renders it inappropriate for applications where several primer-template mismatches need to be considered.

Overall, all three methods achieved high predictive performances on the IGHV data set. Although the predictive performance of *FE*_*Y*_ was surprisingly high, the considerably lower performance of *FE*_*s*_ indicates that the free energy of annealing by itself lacks robustness. In contrast to DE, which estimates the efficiency of polymerase elongation according to the impact of position- and base-specific effects in the 3′ region, TMM considers only the position of 3′ mismatches. The following two observations could explain why the consideration of base-specific effects did not provide an advantage over TMM, although their influence is extensively described in the literature. First, none of the primers contained in the IGHV data set displayed terminal nucleotides other than G or C (Table 3). Second, since base-specific differences in amplification efficiencies have only been reported for qPCR^8^, these difference may simply not be observable with data from gel electrophoresis. Additionally, the present data (Table 2 and Fig. 3) suggest that even simple stringent approaches can be used to ensure high rates of amplification, for example, requiring free energies less than −10 kcal/mol or allowing at most three mismatches.

In order to select a suitable prediction model, its field of application should be carefully deliberated. For example, for multiplex primer design, false positive predictions should be avoided at all costs because they may preclude the amplification of templates that are not redundantly covered. False negative predictions, on the other hand, are much more tolerable. Our analysis suggests that high-specificity classifiers such as *TMM*_*s*_ or *DE*_*s*_ are most appropriate in this scenario. In multiplex scenarios where it is not necessary to amplify all templates, smaller primer sets can be designed by choosing a model with greater sensitivity.

Although models that estimate the likelihood of amplification should be an integral part of rational primer design approaches, there are few available models for this task. The lack of publicly available PCR data is not only a limiting factor for model development but also for improving our understanding of the molecular characteristics that govern PCR amplification. Only when enough data are available will it be possible to devise more comprehensive models that consider all relevant properties concerning primers, templates, and their interaction. Here, we presented a novel PCR data set on which basis we developed TMM, a model for predicting the PCR amplification status, which is freely available via openPrimeR (http://openprimer.mpi-inf.mpg.de/).

## Materials and Methods

### Template design and PCR measurements

We cloned 47 heavy chain fragments from naive B cells into pCR4-TOPO-vector backbones. Each fragment comprises a different functional IGHV gene with the complete leader (L) region, the complete V region and a short part of the constant region. The individual V genes served as representative templates for two different IGHV-specific primer sets. Set 1 is a set of 16 forward primers that was recently designed using openPrimeR^25^, while Set 2 consists of 4 forward primers that were described previously^26^. We performed three independent PCR reactions for each of the 20 primers on all 47 templates with the same IgM constant region-specific reverse primer (GGTTGGGGCGGATGCACTCC)^30^. All primers used in the experiments are listed in Table 3. PCRs were performed in 25 µL reactions with 2U/rxn Platinum Taq (Thermofisher), 0.2 µM forward and reverse primer, 0.2 mM dNTPs, 1.5 mM MgCl_2_, and 6% Kb extender under the following cycling conditions: 2 min initial denaturation at 94 °C followed by 25 cycles of 30 s at 94 °C, 30 s at 57 °C (Set 2) or 55 °C (Set 1), and 55 s at 72 °C. The expected 600–700 bp fragments were visualized on a 2% agarose gel supplemented with SYBR Safe (Thermofisher) and documented with the BioRAD Gel Doc^TM^ XR + Imaging system.

### Data set construction

Template sequences were retrieved by Sanger sequencing and annotated with IgBlast^31^. Every considered PTP $i\in {\mathbb{N}}$ was assigned a label *y*_*i*_ ∈ {*Amplified*, *Unamplified*} based on the evaluation of gel electrophoresis by five persons. Each of the five reviewers visually inspected the gels and independently classified the amplification status. If a band was visible in a gel, the corresponding measurement was labeled as *Amplified* and otherwise as *Unamplified* (Fig. 1). The following procedure was used to identify *y*_*i*,*j*_, the label of PTP *i* according to reviewer *j* ∈ {1, …, 5} from a set of triplicate measurements. If at least two of three measurements were labeled as *Amplified*, *y*_*i*,*j*_ was set to *Amplified*. Otherwise, *y*_*i*,*j*_ was set to *Unamplified*. Let *n*_*i*,*A*_ = |{*y*_*i*,*j*_|*y*_*i*,*j*_ = *Amplified*}| and *n*_*i*,*U*_ = |{*y*_*i*,*j*_|*y*_*i*,*j*_ = *Unamplified*}| indicate the number of times that PTP *i* was labeled as *Amplified* or *Unamplified*, respectively. By setting

$${y}_{i}=\,\{\begin{array}{c}Amplified,\,\text{if}\,\,{n}_{i,A} > {n}_{i,U}\\ Unamplified,\,{\rm{o}}{\rm{t}}{\rm{h}}{\rm{e}}{\rm{r}}{\rm{w}}{\rm{i}}{\rm{s}}{\rm{e}}\end{array}$$

we labeled PTP *i as Amplified* only if the majority of reviewers had labeled the PTP as *Amplified*.

We used openPrimeR to enrich the PCR data with physicochemical properties relating to primers and PTPs. The most likely binding mode for every PTP was identified by selecting the binding conformation minimizing the number of mismatches. Since the exact annealing site of primers is uncertain for PTPs subject to many mismatches, we excluded PTPs with more than 12 mismatches. This reduced the size of the data set from 940 to 908 observations. Based on the determined binding conformation, we derived further properties such as the position of primer-template mismatches. The free energy of annealing Δ*G* was computed with OligoArrayAux^32^ using temperatures of 55 °C and 57 °C for PTPs from Set 1 and Set 2, respectively. Additionally, the following primer-specific properties were computed: primer length, extent of GC clamp, GC ratio, melting temperature, number of repeats/runs, free energy of secondary structures, and self-dimerization.

For model development purposes, we split the data set into three distinct parts (Table 8). To obtain an independent data set for the selection of classifier cutoffs, 25% of the observations were randomly sampled for inclusion in the validation set. We randomly selected 50% of the remaining observations for inclusion in the training data set, which was used for forming a supervised learning model, and the remainder for inclusion in the test data set, which was used for evaluating model performance.

**Table 8:** Distribution of data set labels.

Data set	*N*	*N* (*y*_*i*_ = *Amplified)*	*N* (*y*_*i*_ = *Unamplified)*
Full	908 (100%)	382 (42.1%)	526 (57.9%)
Validation	227 (25%)	96 (42.3%)	131 (57.7%)
Training	454 (50%)	197 (43.4%)	256 (56.6%)
Testing	227 (25%)	92 (40.5%)	135 (59.5%)

The total number of observations *N* and their labels *y* are shown for the full data set and the constructed subsets for validation, training, and testing.

### Feature encoding

In order to investigate the impact of 3′ terminal mismatches, we implemented several encodings, which are illustrated in Fig. 2. The mismatch feature vector *z* ∈ {0, 1}^6^ relies on a binary encoding to indicate whether a mismatch was identified at the *j*-th position in the 3′ hexamer via

$${z}_{j}=\{\begin{array}{c}1,\,{\rm{i}}{\rm{f}}\,{\rm{t}}{\rm{h}}{\rm{e}}{\rm{r}}{\rm{e}}\,{\rm{i}}{\rm{s}}\,{\rm{a}}\,{\rm{m}}{\rm{i}}{\rm{s}}{\rm{m}}{\rm{a}}{\rm{t}}{\rm{c}}{\rm{h}}\,{\rm{a}}{\rm{t}}\,{\rm{p}}{\rm{o}}{\rm{s}}{\rm{i}}{\rm{t}}{\rm{i}}{\rm{o}}{\rm{n}}\,j\,{\rm{i}}{\rm{n}}\,{\rm{t}}{\rm{h}}{\rm{e}}\,3{\rm{^{\prime} }}\,{\rm{h}}{\rm{e}}{\rm{x}}{\rm{a}}{\rm{m}}{\rm{e}}{\rm{r}}\\ 0,\,{\rm{o}}{\rm{t}}{\rm{h}}{\rm{e}}{\rm{r}}{\rm{w}}{\rm{i}}{\rm{s}}{\rm{e}}\end{array}.$$

Here, *j* ∈ {1, 2, … 6} identifies the 3′ hexamer position such that *j* = 1 indicates the first position in the 3′ hexamer and *j* = 6 indicates the 3′ terminal position. To explicitly model the augmenting effect of co-occurring mismatches in the 3′ hexamer^8^, the total number of 3′ hexamer mismatches was encoded as ${X}_{N}=\sum _{j}{z}_{j}$.

Since positions closer to the 3′ terminus deteriorate PCR efficiency to a greater degree^5,6,12–15^, we encoded the 3′ hexamer mismatch closest to the 3′ terminus by setting

$${i}_{X}=\{\begin{array}{c}\mathop{max}\limits_{{\rm{j}}\in \{1,\ldots ,6\}}\{j|{z}_{j}=1\},\,{\rm{i}}{\rm{f}}\,{X}_{N}\ne 0\\ 0,\,{\rm{o}}{\rm{t}}{\rm{h}}{\rm{e}}{\rm{r}}{\rm{w}}{\rm{i}}{\rm{s}}{\rm{e}}\end{array}.$$

For example, a primer without 3′ mismatches has *i*_*X*_ = 0, while a primer exhibiting mismatches at positions 4 and 6 in the 3′ hexamer has *i*_*X*_ = 6.

### Logistic regression models

We used multivariate logistic regression models in order to investigate the influence of individual features on the template amplification status. Logistic regression is a commonly used approach for problems with categorical outcomes. In this case, we would like to estimate the amplification status *y*_*i*_ ∈ {*Amplified*, *Unamplified* }. Let *p* = Pr(*y*_*i*_ = *Amplified*) denote the probability that a template is amplified and let $\hat{p}$ indicate the corresponding estimated likelihood. Further, let *β*_0_ indicate the model intercept and let *β*_*i*_ with $i\in {\mathbb{N}}$ indicate the weight associated with the *i*-th feature *x*_*i*_. Then the logistic regression model can be formulated as

$$\mathrm{ln}\,\frac{\hat{p}}{1-\hat{p}}={\beta }_{0}+{\beta }_{1}{x}_{1}+\ldots +{\beta }_{n}{x}_{n}.$$

Due to the small number of evaluated primers, only terms relating to PTPs were considered as features for the logistic regression models. The logistic regression models *LR*_1_ and *LR*_2_ were used for studying feature importance. While *LR*_1_ was defined using the mismatch feature vector *z* ∈ {0, 1}^6^, the number of mismatches in the 3′ hexamer (*X*_*N*_), and the free energy of annealing Δ*G*, *LR*_2_ additionally included the terms *i*_*x*_ and Δ*Gi*_*X*_ in order to correct for the association between Δ*G* and *i*_*X*_.

For the definition of a logistic regression model estimating the probability of amplification, we formulated TMM by performing feature selection using backward stepwise selection. This process was guided by the AIC^33^, which is defined as

$$AIC=2\,k-2\,\mathrm{ln}(\hat{L})$$

where *k* is the number of model parameters and $\hat{L}$ indicates the maximum value of the likelihood function. Starting from a model trained on the *LR*_2_ features in the validation set, variables were iteratively eliminated in order to minimize the AIC, thereby ensuring that the final model obtains the best possible fit at the lowest possible complexity.

### Further models and classifiers

In addition to TMM, we considered two additional approaches for predicting template amplification status: FE and DE. FE was selected as baseline model because it relies solely on the free energy of annealing Δ*G*. The model DE is the thermodynamic model of DECIPHER^8^, which considers the impact of mismatches on the efficiency of polymerase elongation. Since all models provide quantitative outputs, we transformed them to classifiers in the following manner. For FE, we applied the classification rule

$$f(x)=\{\begin{array}{c}Amplified,\,{\rm{i}}{\rm{f}}\,{\rm{\Delta }}{\rm{G}}({\rm{x}}) < {\rm{\Delta }}{{\rm{G}}}_{{\rm{c}}}\\ Unamplified,\,{\rm{o}}{\rm{t}}{\rm{h}}{\rm{e}}{\rm{r}}{\rm{w}}{\rm{i}}{\rm{s}}{\rm{e}}\end{array}$$

where Δ*G*(*x*) is the free energy of annealing of sample *x* and Δ*G*_*c*_ is a cutoff on the free energy of annealing. For DE, we performed classification by applying a cutoff η_c_ on the PCR efficiency *η*(*x*) computed by DECIPHER:

$$f(x)=\{\begin{array}{c}Amplified,\,{\rm{i}}{\rm{f}}\,\eta ({\rm{x}}) > {\eta }_{{\rm{c}}}\\ Unamplified,\,{\rm{o}}{\rm{t}}{\rm{h}}{\rm{e}}{\rm{r}}{\rm{w}}{\rm{i}}{\rm{s}}{\rm{e}}\end{array}$$

Finally, for TMM, we applied a cutoff ${\hat{p}}_{c}$ on the estimated likelihood of amplification $\hat{p}$:

$$f(x)=\{\begin{array}{c}Amplified,\,{\rm{i}}{\rm{f}}\,\hat{{\rm{p}}}({\rm{x}}) > {\hat{{\rm{p}}}}_{{\rm{c}}}\\ Unamplified,\,{\rm{o}}{\rm{t}}{\rm{h}}{\rm{e}}{\rm{r}}{\rm{w}}{\rm{i}}{\rm{s}}{\rm{e}}\end{array}$$

We selected two cutoffs for each approach: one cutoff ensuring an empiric specificity of at least 99% (denoted by *s*) and another cutoff maximizing Youden’s index *Y* = sensitivity + specificity − 1. For FE and DE, which did not require model training, we selected optimal cutoffs by maximizing the two criteria on a data set containing training and validation observations. For TMM, cutoffs were chosen by performing 10 runs of 5-fold cross validation on the validation data set. Finally, all model and classifier performances were determined on the independent test set.

## Data Availability

The IGHV data set is available via openPrimeR and figshare (10.6084/m9.figshare.6736175 for the raw PCR data, 10.6084/m9.figshare.6736232 for the feature matrix). The code pertaining to the analyses is available at http://www.github.com/matdoering/openPrimeR-User/tree/master/src/primerAmplification. The IGHV data set was annotated using the following code: http://www.github.com/matdoering/openPrimeR/tree/master/data-raw/RefCoverage.R.
